# Blunt liver trauma: a descriptive analysis from a level I trauma center

**DOI:** 10.1186/s12893-018-0369-4

**Published:** 2018-06-19

**Authors:** Ibrahim Afifi, Sheraz Abayazeed, Ayman El-Menyar, Husham Abdelrahman, Ruben Peralta, Hassan Al-Thani

**Affiliations:** 1 Department of surgery, Trauma Surgery section, Hamad General Hospital (HGH), Doha, Qatar; 2 Department of surgery, HGH, Doha, Qatar; 3 Clinical Research, Trauma & Vascular Surgery, HGH, Doha, Qatar; 4 Clinical Medicine, Weill Cornell Medical College, PO Box 3050, Doha, Qatar

**Keywords:** Blunt hepatic trauma, Liver injury grades, Management, Complications, Outcome

## Abstract

**Background:**

We aimed to review liver injury experience in a level 1 trauma center; namely clinical presentation, grading, management approach and clinical outcomes.

**Methods:**

It is a retrospective analysis to include all blunt liver injury patients who were admitted at the Level 1 trauma center over a 3-year period. Data were compared and analyzed based on the liver injury grades and management approaches.

**Results:**

Blunt liver injury accounted for 38% of the total blunt abdominal trauma cases with a mean age of 31 ± 13 years. Liver injury grade II (44.7%) was most common followed by grade I (28.8%), grade III (19.1%), grade IV (7.0%) and grade V (0.4%). Blood transfusion was more frequently required in patients with grade IV (*p* = 0.04). Out of 257 patients with blunt liver trauma, 198 were initially treated conservatively, that was successful in 192 (97%), whereas it failed in 6 (3%) patients due to delayed bleeding from hepatic hematoma, associated splenic rupture and small bowel injury which mandate surgical intervention. Fifty-nine patients (23%) underwent emergent surgery in terms of packing, resection debridement, left lobe hepatectomy and splenectomy. Hepatic complications included biloma, pseudoaneurysm and massive liver necrosis. Subanalysis of data using the World Society of Emergency Surgery (WSES) classification revealed 19 patients were categorized as a WSES grade IV who needed surgical intervention without having an initial computerized tomography scanning. The overall mortality was 7.8% which was comparable among the conservative and operative group.

**Conclusions:**

In our center, low grade liver injury in young males prevails. NOM is successful even for high graded injuries. All conservatively treated patients with high-grade liver injuries should be closely monitored for signs of failure of the non-operative management. Introducing the new WSES classification makes clear how is important the hemodynamic status of the patients despite the lesion. However, further larger prospective and multicenter studies are needed to support our findings.

## Background

Over the past two decades, treatment of blunt hepatic injuries has been changed dramatically. A shift has occurred from the operative management (OM) emphasizing non-resection techniques and packing in the 1980s to selective non-operative management (NOM) in the 1990s and currently to NOM with selective operative management that relies on the computerized tomography (CT) scan findings [1]. Abdominal CT scan is an appropriate modality for the accurate diagnosis and grading of liver injuries in hemodynamically stable patients and is considered useful to guiding the management approach [2]. Beside injury grading, CT scan detects active bleeding (i.e., blush, contrast extravasation and venous phase), pseudoaneurysm which is a common cause of failure to NOM, and associated intraperitoneal injuries and also it quantifies the associated hemoperitoneum [3]. The utility of intervention with angioembolization supports liver injury management either primarily as an adjunct to NOM or immediately post packing in the ‘sandwich technique’ approach in OM [2, 4]. This change in the management has several potential benefits in terms of early hospital discharge, cost-effectiveness, and minimization of nontherapeutic celiotomies, intra-abdominal complications, and blood transfusion [5]. Selective NOM of blunt hepatic injury is associated with less mortality when compared to operative therapy [6–9]. The current literature supports NOM for all grades of blunt liver injury in hemodynamically stable adults, but inconsistency still exists in terms of the efficacy, patient selection, and management of high-grade injury [7, 10–13]. Operative management is usually considered immediately for the hemodynamically unstable patients with extensive injuries or selectively to treat liver injury-associated complications [14–16]. To date, the OM have limited approaches such as perihepatic packing, resection-debridement, and selective vascular ligation [7, 15]. Improved survival after liver trauma could be attributed to the decline in major venous injuries requiring operative intervention, as well as the widely used hemorrhagic control by angioembolization as adjunct to NOM, improved outcomes with venous injuries and hepatic packing [16].

Hemodynamic instability is the primary indication for OM [14, 17, 18]. The objective of this study is to examine the prevalence, clinical presentation, management and outcomes of patients sustained blunt liver injuries in a small rapidly developing country in the Middle East.

## Methods

It is a retrospective chart review study that included all blunt abdominal trauma patients with liver injuries who were admitted and treated at the national Level 1 trauma center in the state of Qatar, from June 2011 and June 2014. Data were extracted from the trauma registry, which is a mature database, in existence since 2007 that is a participant in both the National Trauma Data Bank (NTDB) and the Trauma Quality Improvement Program (TQIP) of the American College of Surgeons-Committee on Trauma (ACS-COT). Inclusion criteria included all blunt liver trauma patients with complete relevant data in adults of both genders (age ≥ 18 years). Patients sustained penetrating abdominal injuries and those who were declared dead at the scene or on arrival were excluded.

Collected data included demographics (age, gender, nationality), mechanism of injury, associated injuries, patient characteristics, Glasgow Coma Score (GCS), Injury severity score (ISS), vital signs (admission blood pressure, heart rate, respiratory rate), laboratory (hemoglobin, hematocrit, platelet count, white blood count, base deficit, International normalized ratio, Alanine Aminotransferase, Aspartate Aminotransferase, and Alkaline Phosphatase) and computed tomography (CT) findings when available, blood transfusion use, blood products transfused, severity of liver injury, ED disposition, management approach and outcomes including length of hospital and Trauma intensive care (TICU) stay,ventilator days, complications and mortality. All patients are resuscitated according to the advanced Trauma Life Support guidelines (ATLS) guidelines [19]. The severity of liver injury is reported using the organ injury scale (OIS) proposed by the American Association for Surgery of Trauma (AAST) and were graded as I-VI based on abdominal CT scan and/or intra-operative findings [20]. Focused Assessment with Sonography for Trauma (FAST) is utilized in the early assessment of all patients to detect the presence or absence of hemoperitoneum. CT scan abdomen was performed in hemodynamically stable patients. Non-operative management was considered in patients who met the following criteria: hemodynamic stability, transfusion of 2 packed red blood cells in relation to liver injury, absence of signs of peritonitis and other abdominal injuries that demand immediate surgery. All patients with high grade liver injury were admitted to the TICU for closed observation and follow-up. This enables early identification of hemodynamic deterioration as indicated and conformed by a significant drop of hemoglobin levels (i.e., acute drop in hemoglobin to a level of 7–8 g/dL).

Planned repeating of abdominal CT scan for higher grades liver injury was not a routine procedure during the study period. On the other hand, hemodynamically unstable patients were candidates for either immediate angioembolization when feasible or surgical treatment depending upon the severity of hepatic injury and degree of instability.

The failure of NOM was defined as need to resort to operative management after a period of watchful observation in TICU weather the reason was related to the liver or associated injuries or demonstration of re-bleeding and need for late angioembolization.

Our protocol for management of liver trauma patients begins with the standard assessment of trauma patients based primarily on their hemodynamic stability status, where unstable patients with positive FAST are directly shifted for operative management (i.e., exploratory laparotomy). On the other hand, stable patients and/or rapid responders to fluid boluses are directed to NOM; starting with an immediate IV enhanced CT scanning of the abdomen within 30–60 min after arrival and subsequent management according to the CT findings. Stable patients with positive FAST are directed to CT scan while the operative theater were kept ready for possible intervention if patient develops hypotension or there is an evidence of active bleeding on abdominal CT and not tolerate a delay to try angioembolization.

Indications of angioembolization included: 1-Presence of arterial blush in the CT scan for either the liver or spleen, if the patient is hemodynamically stable throughout the initial assessment.

2-Post-operative for unstable high-grade liver injury, where damage control perihapatic packing is used then angioembolization before return to OR for definitive repair and closure (Sandwich technique).

3-Evidence of arterial bleeding from associated other injuries (i.e., pelvic fractures, lumbar vessels, or other solid organ injuries).

4-Treatment for pseudoaneurysm and arteriovenous fistula that are diagnosed upon follow up and for rebleeding or hemobilia which occur in some patient as a complication of high grade liver injuries during follow up.

This study was conducted in line with the STROBE checklist (https://www.strobe-statement.org/index.php?id=strobe-home), and has been registered at: http://www.researchregistry.com: Researchregistry3211.

### Statistical analysis

Data were presented as proportions, mean ± standard deviation or median as appropriate. Shapiro–Wilk test was the test of normality for continuous variables, whenever applicable. Sample size was not determined priori as we intended to include all liver injury cases during the study period. Blood products transfused, injury severity, and outcomes were compared according to hepatic injury grades using the one-way ANOVA test for continuous variables and Pearson chi-square test for categorical variables. Injury characteristics and outcome of patients with blunt liver injury were also analyzed according to treatment (non-operative versus operative management) using the Student’s t test for continuous variables and Pearson chi-square test for categorical variables. The Fisher’s exact test was used, if the expected cell frequencies were below 5. Two tailed *p*-values < 0.05 were considered to be significant. Data analysis was carried out using the Statistical Package for Social Sciences version 18 (SPSS Inc., Chicago, IL, USA).

## Results

### Patients’ demographics and clinical characteristics

Blunt abdominal trauma constituted 15% of the total trauma admissions of each year in our center. During a 3-year study period, there were 257 patients with blunt liver injury (84% males) admitted to the level 1 trauma center which accounted for 38% of the blunt abdominal trauma cases. The mean age of patients was 31 ± 13 years. The most frequent mechanism of injury was motor vehicle collisions (37%), followed by fall from height (15.2%) and pedestrian struck (7.4%).

**Fig. 1:**
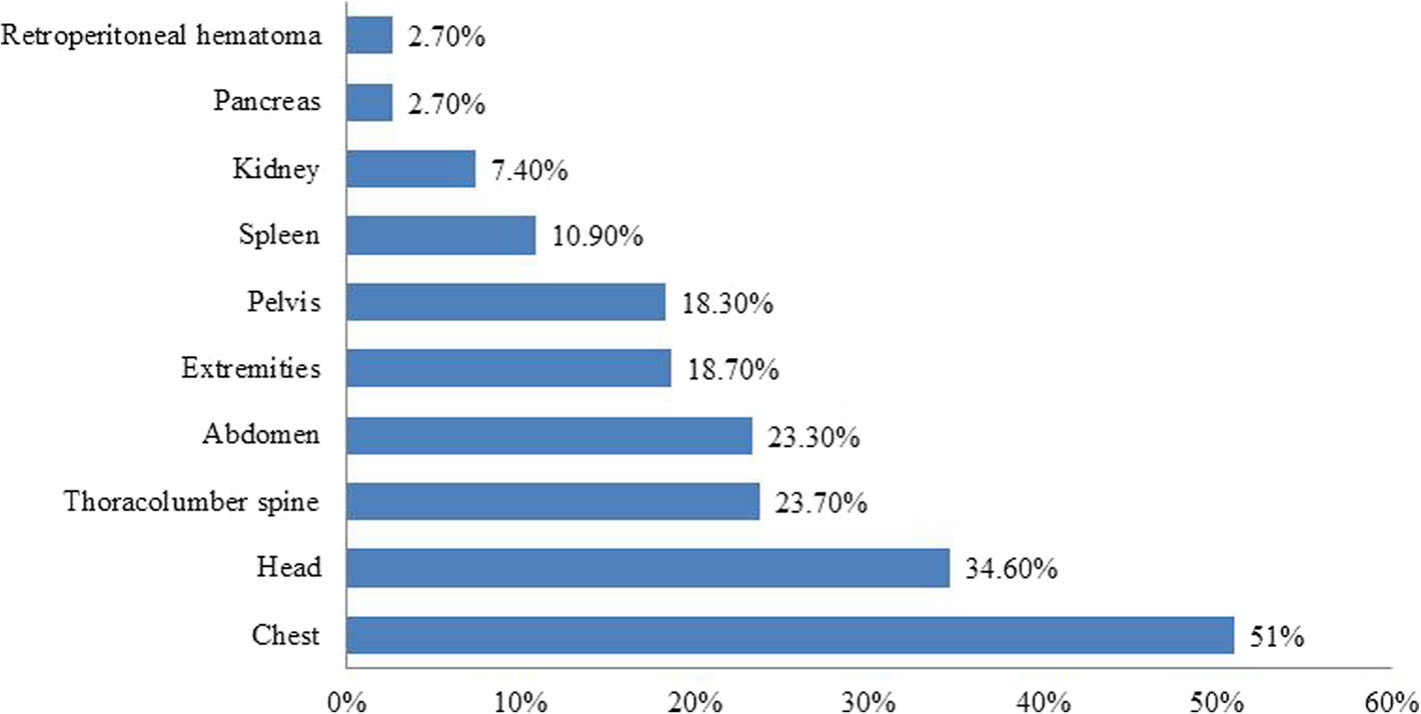
Associated injuries in blunt liver trauma

Table 1 shows demographics, clinical presentation and intra-operative findings of blunt liver injury. The most commonly involved extra-abdominal injuries were chest (51%), head (34.6%), thoracolumbar spine (23.7%) and pelvis (18.3%) (Fig. 1). The frequently associated abdominal injuries were spleen (10.9%), kidney (7.4%), retroperitoneal hematoma (2.7%), and pancreas (2.7%). FAST test was positive in 42 cases. Hollow viscus injuries occurred in a total of 23 patients (8.9%), of which small bowel, large bowel and stomach injuries were identified in 11, 9 and 3 patients, respectively. The mean ISS and GCS were 19.6 ± 11.4 and 13 ± 2.0, respectively. Low GCS and hemodynamic instability were indications for intubation in 57 patients (22.2%). Blood transfusion was required in 27.2% of cases.

**Table 1:** Demographics, clinical presentation, laboratory, intra-operative findings and complications

Variables	Value	Variables	Value
Age; years (Mean ± SD)	31 ± 13	CT abdomen findings	177 (68.9%)
Males; n (%)	216 (84.0%)	Laceration	99 (38.5%)
Nationality		Liver contusion	81(31.5%)
Qatari; n (%)	46 (18.5%)	Perihepatic fluid	14 (5.4%)
Non-Qatari; n (%)	202 (81.5%)	Blush	8 (3.1%)
History of liver disease	2 (0.8%)	Shattered liver	1 (0.4%)
Vital signs		Intra-operative findings	
Systolic blood pressure (mmHg)	114.5 ± 20.1	Laceration	9 (3.5%)
Diastolic blood pressure (mmHg)	69.7 ± 14.9	Hematoma	6 (2.3%)
Heart rate (beat/min)	97.4 ± 22.8	Lobectomy	1 (0.4%)
Oxygen saturation % (Mean ± SD)	97.7 ± 4.6	Active bleeding	2 (0.8%)
Laboratory parameters		Mesenteric tear	3 (1.2%)
Hemoglobin g/dL	13.0 ± 2.3	Complications	
Hematocrit % (Mean ± SD)	39.8 ± 6.6	Pneumonia	43 (16.7%)
White blood cell count 10^9^/L	16.1 ± 6.8	Sepsis	26 (10.1%)
Platelet count 10^9^/L	257 ± 79	ARDS	9 (3.5%)
Base excess mEq (Mean ± SD)	−4.8 (−22.4–7.0)	Wound dehiscence	4 (1.5%)
International normalized ratio ((Mean ± SD)	1.15 ± 0.71	Coagulopathy	2 (0.8%)
Alanine Aminotransferase U/L	172 (7–1534)		
Aspartate Aminotransferase U/L	175.5 (10–996)		
Alkaline Phosphatase U/L	69.7 ± 37.4		
Glasgow coma score (Mean ± SD)	12.7 ± 4.2		
Injury severity score(Mean ± SD)	19.6 ± 11.4		
Ethanol intake; n (%)	34 (13.2%)		
Ethanol level mmol/L (Mean ± SD)	39.3 ± 18.5

Liver injury grading and major findings on initial abdominal CT scan are summarized in Table 1.

Table 2 demonstrates the blood transfused based on liver injury grades. The need for 1–2 units of packed RBCs was more frequent in patients with grade I and II liver injuries. While, significantly more number of PRBCs were transfused to patients with grade IV liver injuries (*p* = 0.02). Massive transfusion defined by transfusion of more than 10 units of packed RBCs was needed in 38.9% of grade IV injuries and less commonly in the lower grades (I-III) due to associated non-hepatic injuries.

**Table 2:** Blood products transfused based on liver injury grades

	Grade I (*n* = 74)	Grade II (*n* = 115)	Grade III (*n* = 49)	Grade IV-V (*n* = 19)	P
Packed RBC unit					
Not transfused	55 (74.3%)	83 (72.2%)	40 (81.6%)	9 (47.3%)	0.02 for all
1–2	7 (9.5%)	12 (10.4%)	2 (4.1%)	1 (5.6%)	
3–6	4 (5.4%)	7 (6.1%)	4 (8.2%)	2 (11.1%)	
7–10	1 (1.4%)	6 (5.2%)	1 (2.0%)	0 (0.0%)	
> 10	7 (9.5%)	7 (6.1%)	2 (4.1%)	7 (38.9%)	
Fresh frozen plasma unit					
Not transfused	60 (81.1%)	93 (80.9%)	43 (87.8%)	9 (50.0%)	0.05 for all
1–2	3 (4.1%)	6 (5.2%)	0 (0.0%)	0 (0.0%)	
3–6	5 (6.8%)	7 (6.1%)	4 (8.2%)	4 (22.2%)	
7–10	0 (0.0%)	3 (2.6%)	0 (0.0%)	2 (11.1%)	
> 10	6 (8.1%)	6 (5.2%)	2 (4.1%)	4 (21%)	
Platelets units					
Not transfused	61 (82.4%)	100 (87.0%)	44 (89.8%)	10 (53%)	0.08 for all
1–2	3 (4.1%)	1 (0.9%)	0 (0.0%)	1 (5.6%)	
3–6	3 (4.1%)	6 (5.2%)	3 (6.1%)	3 (16.7%)	
7–10	0 (0.0%)	1 (0.9%)	0 (0.0%)	1 (5.6%)	
> 10	7 (9.5%)	7 (6.1%)	2 (4.1%)	4 (22.2%)	

A comparison of associated injuries, severity of injury and outcome among different liver injury grades is shown in Table 3. Low GCS, high ISS and mortality rates were comparable among all liver injury grades. Subanalysis of data using World Society of Emergency Surgery (WSES) classification revealed19 patients were categorized as a WSES grade IV who underwent surgical intervention without having initial CT scanning. Table 4 shows the AAST and WSES classification of liver injury.

**Table 3:** Associated injuries, ISS, and blood transfusion in liver injury grades

	Grade I (*n* = 74)	Grade II (*n* = 115)	Grade III (*n* = 49)	Grade IV-V (*n* = 19)	P
Associated injuries					
Head	37 (50.0%)	50 (43.5%)	1 (2.0%)	1 (5.3%)	0.001
Chest	37 (50.0%)	60 (52.2%)	22 (44.9%)	12 (63.2%)	0.45
Pelvis	7 (9.5%)	26 (22.6%)	9 (18.4%)	5 (26.3%)	0.15
Injury severity score ≥ 16	43 (58.1%)	51 (44.3%)	27 (55.1%)	–	0.74
Glasgow coma scale ≤8	18 (24.3%)	24 (20.9%)	5 (10.2%)	1 (5.3%)	0.16
Blood transfusion	19 (25.7%)	32 (27.8%)	9 (18.4%)	10 (52.6%)	0.04
Hospital mortality	9 (12.2%)	9 (7.8%)	2 (4.1%)	0 (0.0%)	0.35

**Table 4:** AAST and WSES classification

Liver injury Severity	AAST		WSES (stable)		WSES (grade IV)
	Grade	n	Grade	n	n
Mild	I-II	189	I	176	13
Moderate	III	49	II	48	1
Severe^a^	IV-V	19	III	14	5

^a^Only 1 case had Grade V, *AAST* American Association for Surgery of Trauma, *WSES* World Society of Emergency Surgery classification

### Management

Table 5 shows the management and outcomes of hepatic injury patients in the study cohort.

**Table 5:** Injury characteristics and outcome in operative and non-operative management

	Non-operative (*n* = 198)	Operative (*n* = 59)	P
Age; mean±SD	31±12	32±16	0.74
Gender (male)	83.5%	86.4%	0.70
Glasgow coma scale < 8	37 (18.7%)	11 (19.3%)	0.91
Injury severity score	17(4–59)	17(5–50)	0.13
Systolic blood pressure < 90 mmHg	15 (7.6%)	7 (12.3%)	0.27
Injury severity score	18.9 ± 11.0	22.0 ± 12.4	0.11
Splenic injury	19 (9.6%)	9 (15.3%)	0.22
Kidney injury	13 (6.6%)	6 (10.2%)	0.35
Pelvic injury	34 (17.2%)	13 (22.0%)	0.39
Liver grade (I-III) AAST	190 (96.0%)	48 (81.4%)	0.001
Liver grade (IV-V) AAST	8 (4.0%)	11 (18.6%)	
FAST	52(29.5%)	29(53.7%)	0.001
CT Abdomen	137(69.2%)	40(67.8%)	0.84
WSES classification			0.03 for all
I	147(74%)	27(46%)	
II	43(22%)	7 (12%)	
III	8 (4%)	6 (10%)	
IV	–	19(32%)	
Medications			
Tranexamic acid	4(3.1%)	1(2.8%)	0.91
Factor VII	10(7.8%)	12(28.6%)	0.001
Fibrinogen	19(14.5%)	17(38.6%)	0.001
Inotropes	19(15.2%)	14(40%)	0.001
Blood transfusion	36 (18.2%)	34 (57.6%)	0.001
Hospital length of stay; median and range	7 (1–323)	13.5 (1–277)	0.23
TICU stay (days);median and range	3(1–41)	7(1–39)	0.01
Hospital mortality	13 (6.6%)	7 (12.1%)	0.17

#### Non-operative management

Out of 198 stable patients, conservative management was successful in 192 (97%); Splenic injury was reported in 19 patients (7.4%) and one patient had mesenteric tear in NOM group. The NOM failed in 6 (3%) patients due to delayed bleeding from hepatic hematoma, associated splenic rupture and small bowel injury which mandate an operation. One patient was successfully managed with angioembolization while the remaining five patients were operated successfully.

#### Operative management

Fifty-nine patients (23%) underwent emergent surgery which included packing (perihepatic and/or intraparenchymal hemostatic packs), resection debridement, left lobe hepatectomy and splenectomy. The primary indications for surgery were hemodynamic instability, peritonism/peritonitis and other associated intra-abdominal injuries that necessitate surgical interventions (Table 5). Operative findings revealed grade III liver injuries in 40 (67.8%), grade IV in 18 (30.5%) patients, and one (1.7%) patient had grade V injury.

### In-hospital complications and outcomes

Overall, pneumonia (16.7%), sepsis (10.1%) and ARDS (3.5%) were the most frequently associated in-hospital complications. Specifically, complications related to liver injury per say include biloma in two cases managed with laparoscopic wash out and drainage. Three cases developed pseudoaneurysm on the NOM group. Massive liver necrosis occurred in one patient after angioembolization and was managed successfully conservatively. The overall mortality rate was 7.8% (20 patients) which was higher but statistically not significant in the OM group (6.6% vs. 12.1%; *p* = 0.17).

Based on the liver injury grades, the mortality was 12.2% in Grade I, 7.8% in Grade II, and 4.1% in Grade III, which is mainly attributed to the severity of associated traumatic brain injuries.

Table 5 demonstrates the injury characteristics and outcome between OM and NOM. The two groups were comparable for severity of injury, associated injuries (spleen, kidney and pelvis) and length of hospital stay. However, the rate of FAST, medications used to control bleeding; AAST grading, WSES classification and TICU length of stay were different in the 2 groups.

## Discussion

This is a large descriptive study from a single center that reports clinical presentations, management and outcomes of blunt liver injuries based on the injury grade, hemodynamic status and management approach. The study showed a higher rate of liver injuries in young males which occurred in more than one third of patients with blunt trauma. Conservative treatment was the option of treatment in more than a three-quarter of cases.

The mechanism of liver injury differs geographically due to socio-demographic and community factors [21, 22]. In the state of Qatar, the rate of blunt poly-trauma associated hepatic injury is high secondary to the increase in the trend of motor vehicles crashes over the recent decades [23].

Earlier studies on blunt hepatic trauma showed an association with extra-abdominal injuries involving chest and head regions as well as fractures of the long bones and pelvis [11].

It has been well established that around 80% of patients with liver injuries can be successfully managed non-operatively [22]. However, this approach could fail in up to 25% of cases due to re-bleeding, bile leak, liver necrosis or secondary sepsis. Our study demonstrated that the vast majority of patients were successfully treated conservatively. Consistent with our findings, an earlier study from Kuwait showed an 80% success rate of NOM in patients who sustained blunt liver trauma and only 4 patients (4%) failed NOM. A recent study from Albania reported a similar rate of successful conservative management (83%) [3]. Another study from Turkey [24] included 300 patients (63% stable and 37% unstable), of them 192 patients treated conservatively and 108 received surgery. In this study, 13% died and the main determinants of mortality were hemodynamic instability on admission and type and grade of liver injury [24].

In our study, conservative treatment failed in six patients mainly due to delayed bleeding from hepatic hematoma, associated splenic rupture or small bowel injury. These findings reflect the improvement of the NOM approach as compared to earlier study by Bernardo et al., [14] where 60.8% of cases were treated non-operatively with a failure rate of 15%. In our series; one patient failed NOM which later successfully treated with angioembolization. Recent literature suggests that NOM in higher grade of liver injury can be considered using selective angioembolization in the absence of active bleeding. Despite the fact that angioembolization is promising adjunct for increasing the success of NOM in blunt liver injury, it could be associated with serious complications such as liver necrosis, secondary infection, liver abscess, bile leak and biloma [25].

In the present study, 23% cases underwent emergent surgery where the major indications for surgery included hemodynamic instability, acute peritonitis and associated other surgically correctable intra-abdominal injuries. Failure of NOM was related mainly to high grade liver injury. Although the failure rate was low, three cases had re-bleeding due to development of pseudoaneurysm. Current reports have suggested the rate of successful NOM to be 60–70% for high-grade liver injuries (i.e. III and above) [26].

Østerballe et al. [27] reported a 4% of pseudoaneurysm on radiological follow-up for 188 patients. The authors observed no correlation between the development of pseudoaneurysm and severity of liver injury; therefore they recommended a follow-up CT angiogram after 4–5 days to rule out such complications. As the high grade injury is the mainstay of failure, we have changed our institutional protocol to repeat the CT scan in patients with higher liver injuries grades with intravenous contrast to pick up early pseudoaneurysm development and to plan angioembolization (coiling) aiming to reduce the rate of NOM failure.

In the present study, complications related to liver injury were very few in terms of biloma in two cases and pseudoaneurysm related rebleeding in three patients. Much less to Carrillo et al. series [28]; who reported biloma of 2.8% in cases with complex blunt hepatic injuries. Bala et al. analyzed 398 patients with liver trauma and identified complications in 16 patients with high grade injury which included biloma and bile leak that was treated with drainage and endoscopic retrograde cholangiopancreatography, while three patients developed re-bleeding from pseudoaneurysm that required angioembolization [29].

Generally, in about 10–20% of severe hepatic injuries, the decision for surgery poses a difficult challenge for surgeons. Non-operative management of high grade liver injuries may carry risk of complication which can be related to the amount of blood transfusion, associated injuries, age and/or liver related complications [30]. The concept of damage control in patients with abdominal trauma is currently a valuable operative approach in unstable patients with liver injury as well as polytrauma [31].

Similar to our findings, few studies have suggested that the need for blood transfusion is lesser in patients who are managed non-operatively than those who underwent surgery [5, 21]. Notably, an earlier study from Egypt reported blood transfusion in 70.5% cases managed non-operatively [21] which is much higher than that of our study (18%). It is worthy to mention that the need of blood transfusion was not dependent on the liver injury alone.

The conservative treatment group showed no significant difference in the length of hospital stay as compared to OM group in our study which is similar to the observation from the Ghnnam et al. study [5].

In our study, the overall mortality rate was 7.8%, and most deaths accounted for significant injuries involving head or chest region and exsanguinating hemorrhage at presentation. The reported mortality rate in hepatic injury patients varies from 9 to 42%, and mostly close to 20% among the admitted patients. However, an earlier study from Saudi Arabia showed a lower rate of motility (3.5%) [5]. The observed high mortality in mild liver injury patients was mainly related to the associated head injury.

Recently, WSES classification for liver injury has been published [16], however, in our center; we still rely on the AAST for grading of solid organ injuries. According to WSES, stable patients should be treated non-operatively in grade I-III whereas WSES grade IV patients should be treated surgically without having initial CT scanning due to patients’ instability. In the present study, there were 189 mild AAST cases, of them 13 patients were classified as WSES grade IV, indicating that surgical intervention was based on the patient instability due to other associated injuries. One-quarter of severe AAST cases was treated surgically and grouped as a WSES IV.

### Limitations

The main limitation of the present study is the retrospective analysis of data which may limit its generalizability in addition to potential selection bias. Patients with incomplete data, prehospital or on arrival death were excluded from the current analysis which may underestimate the blunt liver injury rate. The sample size in the higher grade liver injury groups was small for reliable comparisons. The design of the study makes it difficult to carefully assess the management approach. Moreover, we lack information regarding the time to CT scan and follow-up for patients post-discharge from the hospital.

## Conclusions

Blunt traumatic hepatic injuries are more common in young male population. The vast majority of hepatic injuries are mild (grade I-III) requiring conservative treatment. Therefore, non-operative management of liver injuries is a frequent approach in our practice and has been successfully considered in hemodynamic stable patient. Associated head injury could explain the high mortality in the low grade liver injuries. Major liver injuries (grades IV-V) are relatively infrequent in our cohort. All conservatively treated patients with high-grade liver injuries should be closely monitored in the intensive care unit for the indication of failure of NOM which can be treated further with the help of intervention radiology or operative management. NOM could be successful even in high graded injuries with low morbidity and mortality.

There is a role of routine repeating CT scan for high grade and the utility of angioembolization in the absence of active bleeding to decrease late failures of NOM. Introducing the new WSES classification makes clear how is important the hemodynamic status of the patients despite the lesion. However, further larger prospective and multicenter studies are needed to confirm our findings.
